# A Closer Look at the Potential Mechanisms of Action of Protective Agents Used in the Drying of Microorganisms: A Review

**DOI:** 10.3390/microorganisms13020435

**Published:** 2025-02-17

**Authors:** Charlotte Van Engeland, Benoît Haut, Frédéric Debaste

**Affiliations:** Transfers, Interfaces and Processes (TIPs), Université libre de Bruxelles (ULB), Avenue F.D. Roosevelt, 50 CP165/67, 1050 Bruxelles, Belgium; charlotte.van.engeland@ulb.be (C.V.E.); benoit.haut@ulb.be (B.H.)

**Keywords:** drying, protection mechanisms, bacteria, yeast, protective agents

## Abstract

Yeast, bacteria and sourdough are widely used in our daily lives, yet their drying and storage remains a significant challenge. A variety of techniques are used to improve the resistance of cells to thermal, dehydration, oxidative and osmotic stresses, which can occur at different stages of the process. The addition of protective agents prior to drying is a commonly used method, but the mechanisms that may lead to a change in viability following the addition of these agents, or more generally, the interaction between a protective agent and the drying process, are not yet fully understood. This review outlines seven main potential mechanisms, as highlighted in the literature, which can lead to internal or external modifications of the cells. The mechanisms in question are change of membrane fluidity, accumulation of compounds for osmoregulation, prior osmotic dehydration, prevention of oxidation, coating or encapsulation, enhancement in thermal resistance and change in drying kinetics. A comprehensive explanation of these mechanisms is provided. This review also highlights the connection between the mechanisms and the influence of the stresses occurring during drying and storage, which depend on the drying technique used and the operating conditions, the strains and the protective agents involved, on the importance of the different protection mechanisms. By gaining a deeper understanding of the mechanisms of action of protective agents, strategies to improve the quality of the microorganisms obtained after drying can be developed. One such strategy would be to combine several agents to achieve a synergistic effect.

## 1. Introduction

Drying of microorganisms causes structural and physiological damage to cells, resulting in loss of viability [1,2]. Different stresses can occur during drying, depending on the drying technique used and the operating conditions: thermal stress (heat stress or cold stress), dehydration stress, oxidative stress and osmotic stress [1,3,4,5,6,7]. In general, heat stress is thought to be related to denaturation of macromolecules or cell wall damage [4,8], while inactivation by dehydration seems to affect the cytoplasmic membrane and its fluidity [5,9]. Both external and internal factors contribute to the decrease in viability of bacteria and yeasts [3,10]. External factors include product temperature, drying kinetics and type of drying, while internal causes include damage to cellular structures such as protein denaturation and loss of membrane integrity [2,11,12,13].

Several approaches and techniques are commonly used to improve cell survival during drying and storage. For example, cell pre-adaptation as well as modification of the culture medium, storage temperature or atmospheric conditions are key strategies often considered in attempts to maintain cell viability [3,14]. Consumer-friendly carriers are now commonly used in various forms to improve the viability of microorganisms during drying as well as processability. This reflects a growing awareness of consumer demand for carriers that are free from allergens and as natural as possible. In recent years, the focus has been on healthy, allergen-free protective agents, in line with the clean-label trend [15,16]. The terminology used to describe compounds added prior to drying to provide additional protection during drying and storage is diverse. These compounds are often referred to as protectants, carriers or protective agents. Throughout this paper, the term ‘protective agent’ encompasses all such terms, while the term protective medium is used when more than one protective agent is envisaged. Several recent studies underline the positive impact that protective agents can have on quality, whether they are produced by the microorganisms during culture, or added in post-culture [17,18,19,20]. By optimizing the composition of the protective media and the drying conditions, damage to cellular structures can be minimized [21]. This review focuses on the use of protective agents to maintain viability during drying and storage.

Protective agents are usually categorized according to their type of macro-nutrient—carbohydrates, proteins, amino acids—but not according to their potential protective mechanisms [14,22]. What are the mechanisms that can lead to a change in viability after the addition of protective agents? This question is challenging and still under investigation. The different mechanisms explaining this stability improvement or, more generally, characterizing the interaction between a protective agent and the drying process remain unclear and are still the subject of debate. The existing literature presents a number of different mechanisms that may be involved, and these are discussed here.

In this review, seven main potential mechanisms are presented from the literature. These mechanisms are often related to cell adaptation. Some cells are able to survive desiccation [23], and the addition of certain compounds attempts to reproduce the natural tolerance mechanisms of the cell. Strain-dependent adaptive responses include synthesis of stress proteins, adjustment of membrane composition, accumulation of compatible solutes and energy storage or regulation [24,25,26]. We aim to provide a process-driven review of the various potential mechanisms explaining the observed change in viability following the addition of protective agents, and to characterize their interaction with the drying process. Existing reviews mainly focus on the potential role of each individual molecule, rather than considering the protective mechanisms of agents used to protect cells. This review will also highlight gaps for future studies on this topic.

## 2. Possible Mechanisms After Addition of Protective Agents

The various mechanisms that could explain an improvement in stability or, more broadly, characterize the interaction between a protective agent and the drying process have not been clearly identified, but several hypotheses have been described in the literature. The mechanisms presented in this review are still disputed and are the subject of ongoing research. Seven potential protective mechanisms have been identified in the literature: change of the membrane fluidity, accumulation of compounds for osmoregulation, oxidation prevention, coating or encapsulation, thermal resistance enhancement, prior osmotic dehydration and change in drying kinetics. The aforementioned mechanisms may result in better protection of the microorganisms during the drying phase and/or during prolonged storage. The challenge is to maintain the viability of microorganisms not only during the drying process but also during storage. While the stresses undergone by the cells during storage are different to those experienced during drying, viability can decrease significantly during the storage period [5,27,28], and protective agents can also be beneficial during the post-drying processes.

### 2.1. Change of the Membrane Fluidity

Membrane fluidity may play a critical role in the survival of microorganisms during drying. For example, Meneghel et al. [29] showed that a high freeze-drying resistance of *Lactobacillus delbrueckii* subsp. *bulgaricus* ATCC 11842 appeared to be associated with a high membrane fluidity and a homogeneous distribution of this fluidity. Changes in membrane fluidity are correlated with cell survival and death [30,31]. Membrane fluidity can be altered by several factors. Interactions with the membrane, changes in molecular mobility due to vitrification, and changes in the membrane composition can all cause a change in membrane fluidity. This section briefly outlines how membrane fluidity can be altered and how it relates to viability.

#### 2.1.1. Membrane Interaction: Water Replacement Hypothesis

The structure of the lipid bilayer of cell membranes is maintained by a variety of interactions, including electrostatic bonds of the van der Waals type, interactions between apolar parts and hydrogen bonds, interactions between polar parts [32]. During drying, as the water content decreases, the repulsive hydration force that separates the membranes also decreases, inducing large mechanical stresses in the membranes. The removal of water led to an increase in the van der Waals interaction between the hydrocarbon chains. To some extent, the compressive stress is such that the phospholipids undergo a phase transition from liquid crystalline to gel phase [33]. This transition is characterized by a membrane phase-transition temperature, $T_{m}$. During dehydration, $T_{m}$ increases, and when $T_{m}$ exceeds the operating temperature, a phase change from the liquid crystalline to the gel phase occurs. After rehydration, the membrane returns to its original state and undergoes a transition from the gel to the liquid crystalline phase. These transitions are thought to be responsible for high mortality when the membrane is unstable [31,34,35].

The membrane can be stabilized by the formation of interactions between some protective agents and the membrane [36,37]. The interaction can take place via hydrogen bonds between the hydroxyl groups of the protective agents and the phosphate groups of the membrane [36,38,39,40,41]. These compounds act as a substitute for water molecules and can therefore limit the negative effects of dehydration. In fact, during dehydration, the bonds between water and head groups of phospholipids are gradually lost. The addition of compounds that can replace the hydrogen bonds with the hydroxyl groups prevents the transition of the cell membrane into a gel phase and the formation of hydrogen bonds with the proteins can prevent their denaturation [37]. Indeed, some solutes can lower the membrane phase-transition temperature, $T_{m}$ [34,42,43], and thus the cell membrane remains in liquid phase at lower water content. This mechanism can only be considered for small compounds that can interact with the phospholipid membrane such as monosaccharides, disaccharides and certain polysaccharides, depending on the flexibility of their structure [44]. The importance of this mechanism also depends on the affinity of the protective agent to bind to phospholipids via hydrogen bonding. Indeed, it will depend on the number of groups able to form hydrogen bonds and also the spatial distribution of the OH groups in equatorial and axial positions [45,46,47].

#### 2.1.2. Molecular Mobility: Hindrance of Cytoplasmic Membrane Mobility and Vitrification Theory

The close proximity of phospholipids, as well as the liquid–gel phase transition, can also be limited without any specific interaction with the membrane [33]. Indeed, the addition of certain protective agents increases the osmotic pressure of the solution and thus reduces the stress undergone by the membranes. In addition, the molecular volume of certain compounds can keep membranes at bay [5]. At very low water contents, a further reduction in stress can occur if the compounds vitrify in the intermembrane space. Such vitrification leads to a reduction in molecular mobility and makes it more difficult for the membranes to reduce their spatial distance under compressive stress [5,33,44]. The compounds must be small enough to approach the membrane in question to be able to reduce the compressive stresses and decrease $T_{m}$ [48]. Kumara et al. [49] observed that the size of the added protective agent, in their case sucrose or polysucrose, influences several properties, including the glass-transition temperature ($T_{g}$), the glass fragility, the water retention and the extent of protection provided. The significance of this mechanism therefore depends on the size and glass transition of the added protective agents, but also on the cell size of the microorganisms in question. The vitrification process appears to offer enhanced membrane protection when the protective agent is close to the membranes, thereby increasing its osmotic effect [33].

Bacteria survival seems to be influenced by the glass-transition temperature of the product, $T_{g}$ [49,50,51]. Tantratian and Pradeamchai [51] observed a high cell viability and a reduction in the number of cell injuries after spray-drying with a product having a high glass-transition temperature. However, this was not observed by Siemons et al. [52]. They observed that a high $T_{g}$ of a given matrix does not necessarily lead to an increase in cell viability after drying. They suggested that a glassy state early in the drying process may lead to a decrease in drying kinetics and therefore a longer drying time may be required, which may be detrimental to the cells. For more information on the impact of a change of drying kinetics on viability, see Section 2.7. Perdana et al. [53] also observed that the effect of $T_{g}$ on inactivation due to dehydration stress does not appear to be as simple as increasing $T_{g}$ to provide increased protection after drying. However, it appears that thermal inactivation is reduced when the cells are rapidly enclosed in a glassy matrix [53]. Since water is as a well-known plasticizer, the glass-transition temperature generally decreases with increasing moisture content.

The importance of this mechanism may therefore vary depending on the drying techniques chosen, the residual moisture content and the sensitivity of the strain to thermal and dehydration stress. While the precise influence of vitrification on survival during the drying process is not fully well defined, its influence on storage seems to be more clearly established. A large number of studies have shown that storage after freeze-drying or spray-drying at a temperature below $T_{g}$ appears to improve the survival of several microorganisms [54,55,56]. However, inactivation still occurs when the storage temperature is close to the glass-transition temperature. Several studies have suggested that to achieve an almost complete reduction in molecular mobility, microorganisms should be stored at a temperature of 30–50 °C below the glass-transition temperature [55,57,58]. Even under these storage conditions, the rate of inactivation can be very slow, but it can still be noticeable. It appears that the glass transition is necessary but not sufficient to maintain viability during storage. One possible explanation for the remaining inactivation is that oxidation can still occur because the free radical reaction is not limited by diffusion [59,60]. In conclusion, storage at low moisture content in a glassy matrix in the presence of antioxidant may confer different mechanisms mediating different inactivation stresses.

The first two mechanisms presented, water replacement and molecular mobility, are well described in several reviews [37,61,62,63] that examine the role of sugars in improving viability during drying and storage. These two mechanisms are difficult to prove because they often occur simultaneously and may compete in certain cases. For example, some monosaccharides may stabilize cells by replacing water on the phospholipid bilayer, but at the same time reduce the glass transition [56]. It is therefore difficult to distinguish between the two effects. The stabilization of the bilayer membrane can be attributed mainly to one mechanism or to a combination of the vitrification theory and the water replacement hypothesis [64]. The combination of low molecular weight compounds that can stabilize the membrane by direct interaction with larger molecules that promote a glassy state may be a promising solution [56].

#### 2.1.3. Membrane Composition

To adapt to stress conditions, microorganisms can change the composition, especially the fatty acid composition, of their cell membrane [25,65,66]. Stresses such as heat stress [67], cold stress [68,69], acid stress [69,70] and osmotic stress [30] can induce changes in membrane composition and help maintain the integrity of the cell membrane. Consequently, substances capable of regulating the membrane composition are likely to affect the membrane fluidity and, in some cases, improve the resistance to stress. It should be noted that changes in membrane composition, and therefore membrane fluidity, are stress dependent. In fact, several studies have shown that a rigidification of the membrane could help cells adapt to acid stress [69,70], while an increase in membrane fluidity could help cells to adapt to cold environment [29,69]. It appears that the membrane requires greater rigidity or increased fluidity, depending on the stress to which the microorganisms are exposed, and the strain involved. However, whether the membrane fluidity is too high or too low seems to affect the survival of the microorganisms. Important variations of the membrane fluidity can lead to cellular damages and cell deaths [30,31,71].

The phase-transition temperature from the liquid crystalline to the gel phase depends on the fatty acid composition [72,73]. Tight packing due to long, saturated lipid acyl chains increases van der Waals interactions, which in turn decreases membrane fluidity and increases membrane phase transition. In contrast, shorter, unsaturated chains are less packed and lower the membrane phase-transition temperature. The ratio of unsaturated to saturated fatty acids is then often used to assess the membrane fluidity. However, predicting changes in membrane fluidity is not straightforward because a number of factors influence the membrane fluidity, such as the composition of the lipid head groups, the position of the double bonds and their configuration, as well as the concentration of sterols and proteins [73,74,75]. Cyclopropane fatty acid content has also been found to affect the membrane fluidity, although its effect on membrane fluidity remains unclear. Some have observed that an increase in cyclopropane increases the fluidity [68], while others have observed a decrease in the fluidity following the increase of cyclopropane fatty acids in the membrane [57,76].

There are several approaches to adjust the fatty acid composition, either during the fermentation stages, for example by changing the fermentation temperature or the growth medium [77,78,79], or by adding compounds prior to stress, i.e., fatty acids [80,81] or compounds capable of regulating the phospholipid bilayer and the cell surface membrane [17,82]. The latter is the focus of this review. The addition of oleic acid C18:1 as a protective agent has received much attention in recent years [80,81,83]. Compared with several other protective agents, the addition of C18:1 does not seem to affect the glass-transition temperature and can maintain the membrane integrity and fluidity [80]. The addition of protective agents such as skim milk or a combination of skim milk, trehalose, sorbitol and tyrosine can also affect the membrane composition and therefore may improve the membrane fluidity and resistance to drying [17].

Several studies have shown that increasing the ratio of unsaturated to saturated fatty acids helps to maintain the membrane integrity and increases the freeze-drying resistance of several probacteria such as *L. plantarum* or *L. fermentum* [17,79,80]. These results are consistent with the fact that during drying, the membranes become progressively stiffer with dehydration due to packing of the fatty acyl chain as water is removed [30,84]. Consequently, improving membrane fluidity may prove beneficial in mitigating the stiffening effects of dehydration. It has been observed that less variation in fluidity is beneficial in terms of survival during freeze-drying [30,84]. Studies have shown that specific fatty acids, such as oleic acid and cyclopropane fatty acids (C19cyc11), appear to be more prone to affecting the membrane integrity and fluidity and thus the resistance to drying [17,80]. In contrast, an improved freeze-drying resistance was observed in certain cases when the fluidity of the membrane was low [57,85]. Velly et al. [85] suggested that the mechanical resistance of the membrane is increased by membrane stiffening. In addition, the composition and fluidity of the cell membrane appears to influence the intracellular concentration of osmolytes (see Section 2.2). Indeed, Louedson et al. [76] observed that higher intracellular accumulation of betaine was observed in more rigid membranes. The lower fluidity may reduce the exchange between intracellular and extracellular compartments and promote transport [86]. These contradictory results can be interpreted in a more nuanced way. The improvement in cell viability with membrane stiffening appears to be due to an increase in cyclic fatty acids (CFAs). A recent study by Girardeau et al. [87], based on data from several studies in the literature, highlights that improved survival rate correlates with an increase in the ratio of unsaturated to saturated fatty acids (UFA/SFA) when lactic acid bacteria have a low membrane content of cyclic fatty acids (<10% of the fatty acid composition). At higher cyclic fatty acid content, an increase in CFAs seems to result in an increase in resistance and is no longer not correlated with an increase in unsaturated fatty acids. The composition of the membrane and its influence on fluidity and survival are not yet clearly established [72].

Further research is needed to better understand the effects of membrane composition on fluidity and drying resistance. Given the findings of Girardeau [87], a general explanation that is not strain dependent seems likely. For a more accurate comparison of the different studies, it would be necessary to determine the composition of the cell membrane, its fluidity and its state (liquid or gel phase) before and after drying. As highlighted in the review by Fonseca et al. 2019 [72], in-depth and simultaneous characterization of fatty acid composition, membrane phase transition and fluidity at different process stages should be further investigated to better understand the influence of process and conditions on microorganism responses. We could indeed assume that the effect of membrane composition on membrane fluidity can be expected to vary depending on whether the membrane is in a liquid crystalline phase or a gel phase. Indeed, sterols have been shown to have a stabilizing effect on the membrane. In the liquid crystalline state, the membrane is stiffened by increasing the sterol content, whereas in the gel phase, the addition of sterols increases the fluidity [88]. Further in-depth research is then essential to gain a better understanding of the influence of the membrane composition on its fluidity.

**Figure 1:**
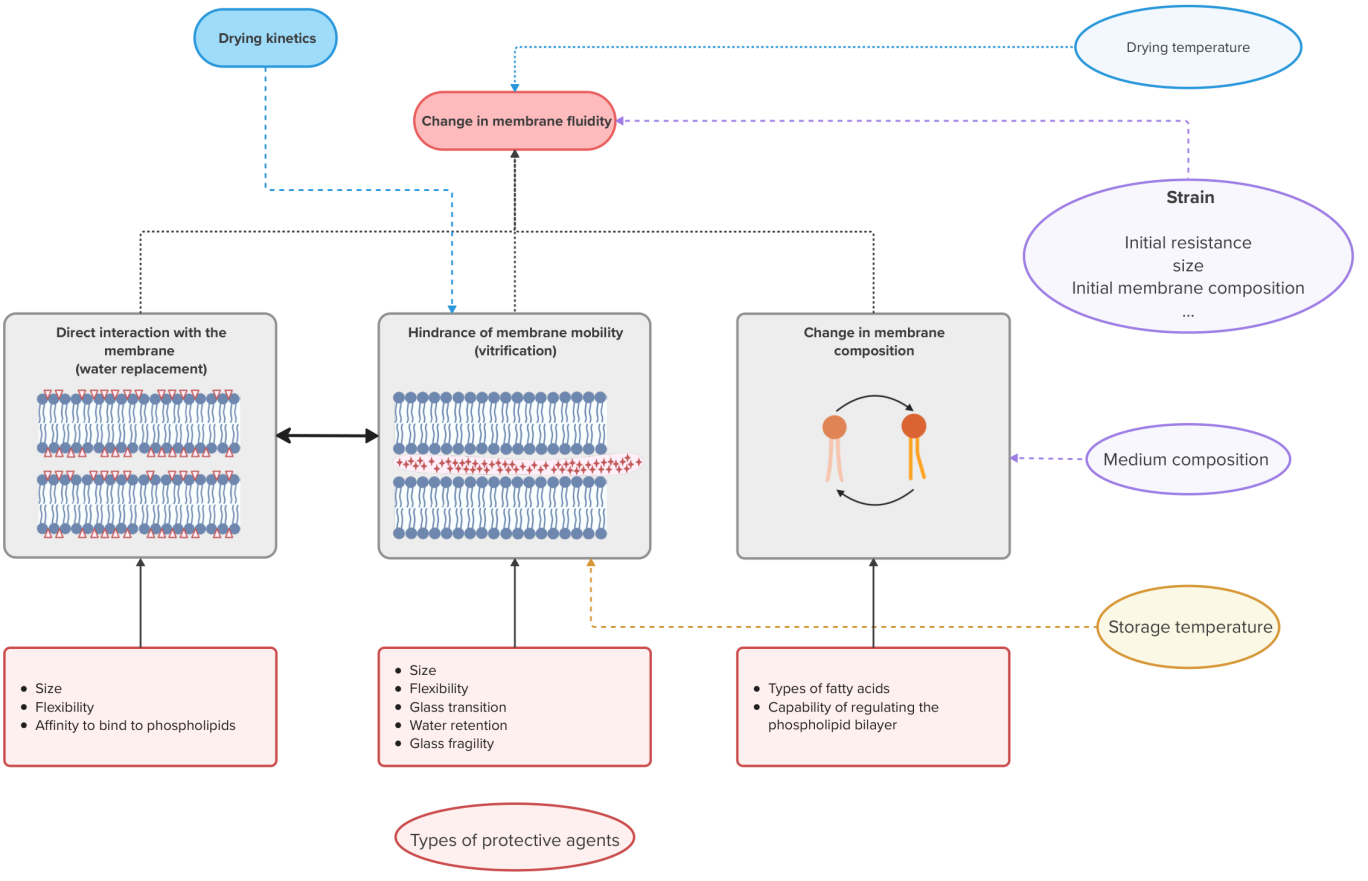
Diagram summarizing the mechanisms of change of membrane fluidity due to direct interactions with the membrane, changes in molecular mobility as a result of vitrification or changes in the membrane composition. The figure contains graphics created in BioRender. Van Engeland, C. (2025) https://BioRender.com/k19a180 (accessed on 3 February 2025). The red symbols in the graphical illustrations of the mechanisms represent a protective agent. The oval boxes represent operating conditions that can be modified.

The mechanisms of cell protection through changes in the membrane fluidity due to direct interactions with the membrane, changes in molecular mobility as a result of vitrification or changes in the membrane composition and the influence of operating conditions on these are graphically summarized in Figure 1.

### 2.2. Accumulation of Compounds for Osmoregulation

The accumulation inside the cells of a number of compounds, such as trehalose, proline, glycine and betaine, appears to provide protection to the cells during drying [19,89,90,91]. This is a natural mechanism that has been observed in microorganisms in response to various stresses [92]. In response to external stresses, microorganisms adjust their production of osmolytes or can accumulate small molecules from the environment [92,93]. These osmolytes are hydrophilic molecules with low molecular weight, classified as kosmotropic, including amino acids [93]. The importance of osmoregulation depends on the metabolic pathway of the cells. Cellular accumulation of trehalose or sucrose can be as high as 20% of the dry weight [90]. The addition of compatible solutes may help to balance the osmotic pressure difference across the cell membrane and then protect the cell from the osmotic stress during dehydration. Vaessen et al. [94] observed that cells in a solution containing trehalose or lactose accumulated these solutes during freeze-drying and spray-drying. Unexpectedly, they observed comparable levels of solutes in *Lactobacillus plantarum* WCFS1 during freeze-drying and spray-drying, despite the significant difference in drying times (ranging from several hours to a few seconds). Previous assumptions suggested that osmolyte uptake would not be a primary factor during short duration drying processes [95,96]. This mechanism has not been extensively studied in cases where a protective agent is added prior to the drying process. In general, this mechanism is studied when osmolytes are added into the growth medium [19,89,91]. Proline and glycine betaine and trehalose are occasionally employed as external excipients that may potentially act as protective compounds [18,97]. However, in these studies, the intracellular concentrations of these solutes were not measured before and after drying. As it is often assumed that accumulation of compatible solutes is unlikely to occur during the relatively short duration of the drying process, few studies have investigated the post-drying accumulation of compatible solutes, such as trehalose, lactose or glycine betaine, when added just before drying [94,98]. These studies have shown that compatible solutes can accumulate during drying, even for short drying times. This highlights the need for additional research on the uptake of compatible extracellular solutes during the drying process when introduced prior to drying, for example by measuring intracellular concentrations of protective agents throughout the drying process.

**Figure 2:**
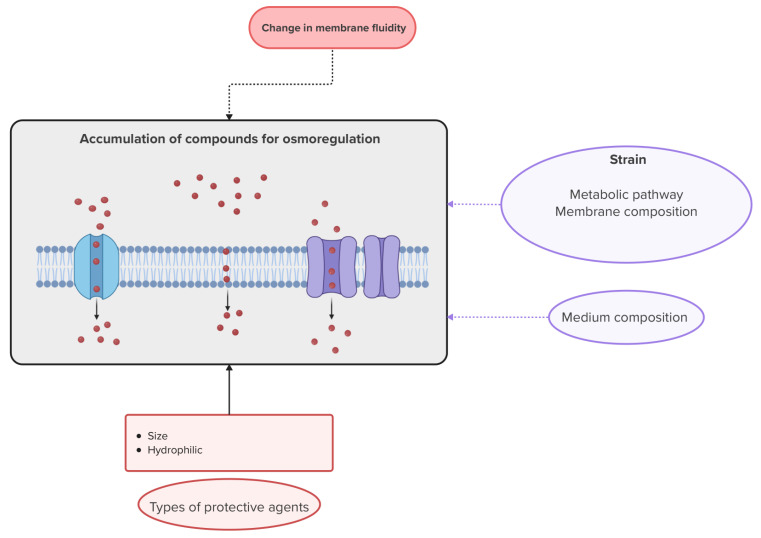
Diagram summarizing the mechanism of compound accumulation for osmoregulation. The mechanism illustration was created with Biorender. Van Engeland, C. (2025) https://BioRender.com/h61f867 (accessed on 3 February 2025). The red circles in the graphical illustration of this mechanism represent a protective agent. The oval boxes represent operating conditions that can be modified.

Osmoregulation and changes in membrane fluidity appear to be complementary mechanisms. Indeed, Louedson et al. [76] observed that intracellular betaine content increases with more rigid membranes. They suggest that intracellular osmolyte may be better retained within cells due to reduced exchanges between the intracellular compartment and the environment resulting from decreased membrane fluidity. In addition, changes in membrane properties could also alter the transport of osmolytes [99]. ABC transport-related proteins, which allow the internalization of osmoprotective organic compounds, are upregulated during stress [100]. Figure 2 provides a graphical summary of the mechanism of accumulation of osmoregulatory compounds and the influence of the operating conditions on this mechanism.

### 2.3. Prior Osmotic Dehydration

The addition of solid protective agents prior to drying can potentially pre-dehydrate the cells of microorganisms by controlling the osmotic pressure exerted across their membranes. Moisture is removed by diffusion rather than evaporation and therefore without phase change [101]. A solid protective agent is often used to facilitate the formation of a powder before drying when the product is too liquid. This mechanism is not often considered in research studies. However, the addition of many compounds can cause osmotic dehydration and even lead to cell death if the osmotic shock is significant [6,102]. By osmosis, water flows through the membrane and at some temperature and pressure, it can be harmful for the cells [6,30]. These mechanisms can have an impact on survival if the protective agents are solid, hygroscopic and in sufficient concentration to cause a significant decrease in moisture content prior to drying. Mille et al. [6,103] lowered the initial moisture content and water activity by mixing cells with wheat flour or casein powder. They observed that good cell survival could be obtained at the end of the drying if the osmotic shock applied via the solid carrier was not too important. A study by Laroche and Gervais [104] has also shown that the initial water activity of a sample is related to the survival of the dried microorganisms when subjected to heat stress. Liu et al. [105] observed that the addition of porous solid carriers could improve the quality of baker’s yeast. They suggested that the addition of solid porous structure contributes to the migration of moisture during the whole drying process and reduces the drying time and thus exposure to stress. In one of our previous works, we have also shown that the addition of a solid carrier, wheat flour, reduces shrinkage during drying and increases the drying rate of yeast pellets [106]. As discussed later in Section 2.7, these changes in the evolution of the physical parameters during drying may also lead to a change in the survival rate. The literature emphasizes that reducing the initial water content and water activity can induce a change in the tolerance of microorganisms to heat and dehydration stress [3,6,104,107].

### 2.4. Oxidation Prevention

**Figure 3:**
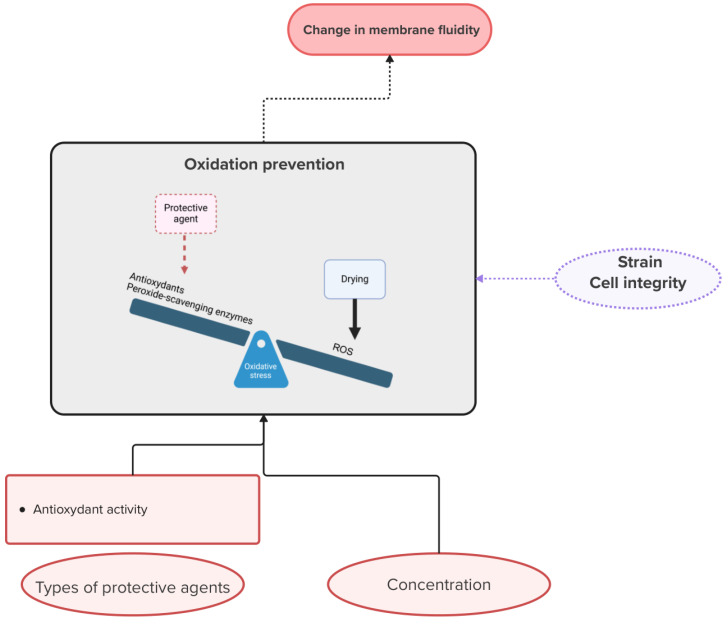
Diagram summarizing the mechanism of oxidation prevention. The mechanism illustration was created with Biorender. Van Engeland, C. (2025) https://BioRender.com/l12o061 (accessed on 3 February 2025). The oval boxes represent operating conditions that can be modified.

Dehydration increases the oxidative state of the cells and the production of reactive oxygen species (ROS), free radicals that can cause oxidative stress when present in excess. An imbalance between ROS and antioxidants or peroxide-scavenging enzymes can lead to peroxidation of lipids, proteins and nucleic acids [108,109,110]. In the study by de Jesus Pereira et al. [111], the dry yeast cells are 10 times more oxidized after dehydration than in the case of fresh cells. This mechanism is particularly relevant for improving the quality and metabolic activity of microorganisms during drying, where the cells are exposed to large volumes of air, and during storage. Indeed, it has been shown that during air drying, cell oxidation can occur and be detrimental to the cells [111,112]. Some compounds such as trehalose, ascorbic acid or ergosterol can reduce lipid peroxidation and improve cell survival [111,112,113,114]. Rodklongtan et al. [112] used ascorbic acid, an antioxidant, to supplement lactose. The antioxidant activity of the ascorbic acid could promote cell survival during spray drying and storage. However, at high concentrations of ascorbic acid, Rodklongtan et al. [112] observed a negative effect on viability. They suggested that increasing the concentration of ascorbic acid reduces the glass temperature and thus the membrane stability. This study demonstrates the importance of the concentration of hypothetical protective compounds on viability and the potential for encountering an opposite effect. It should be kept in mind that the regulation of the antioxidant defense system is quite complex and that the efficacy of the molecules regulating the concentration of free ROS depends on the strain and the cell integrity [108,109,115]. As expected, antioxidants can, in some cases, help alleviate the oxidative stress experienced by cells. By interacting with free radicals to make them less likely to damage cellular components, compounds other than antioxidants may also be effective in reducing oxidative stress. Indeed, trehalose, a disaccharide, has been shown to be able to reduce lipid peroxidation by decreasing the level of ROS [111,114]. Oxidation of membrane lipids may induce change in membrane structure and reduce its fluidity by lowering the ratio of unsaturated to saturated fatty acids [116,117]. Oku et al. [118] showed that the weak interaction of trehalose with the double bonds of unsaturated fatty acids allows it to reduce their oxidation. As discussed in Section 2.1.3, increasing the unsaturated fatty acid content seems to provide additional protection during drying in many cases. However, since unsaturated fatty acids are preferentially oxidized compared with saturated ones, oxidative stress during storage should be more important when unsaturation is used as a protection mechanism. This highlights the possibility of simultaneous positive or negative effects of different mechanisms and the difficulty of dissociating them. A graphical representation of the information presented in this paragraph is provided in Figure 3. This figure illustrates the mechanism, the influence of certain operating conditions on said mechanism and its potential impact on the significance of another mechanism, in this case the modification of membrane fluidity.

### 2.5. Coating or Encapsulation

The formation of agglomerates covering the cells can lead to an improvement in cell viability [119,120,121]. A buffer layer can be formed on the cell surface, creating a physical barrier that could mitigate stresses from heat and dehydration. This mechanism has been reported mainly for polysaccharides and proteins [84,122,123]. Depending on the composition and the mechanical properties of the added compounds, either a rigid hard shell is developed or a skin with elasticity is formed. For example, Khem et al. [124] used whey protein isolate (WPI) and assumed that the hydrophobic interactions between cells and the exposed hydrophobic parts of WPI allow the cells to be embedded in this protective medium. Gelation of protein, polysaccharides or a mixture of protein and polysaccharides is based on protein unfolding and aggregation into a gel network driven by ionic interactions and hydrogen bonds [125,126]. Protein–polysaccharide gels are often used for their efficiency with respect to entrapping water. It should be noted that not all polysaccharides can be used to form a gel phase. For example, xantham gum and λ-carrageenan are considered as non-gelling polysaccharides [127]. The selection of an effective enveloping material with desired physical, mechanical and chemical properties is not straightforward and is based on criteria such as solubility, non-reactivity with the cells, water-holding capacity, emulsifying properties, molecular weight, glass transition, conformation and charge density [125,128,129]. For example, the gelling and film-forming capacities of certain compounds may exert an influence on the size of the agglomerates [123]. The properties of the encapsulating agent, whether alone or in combination, can lead to different properties and, in turn, influence survival [122,128,130]. For example, Afzaal et al. [130] have observed that beads with alginate had a porous structure, while a less porous and more compact structure was observed when inulin and alginate were used together. Further information on encapsulation techniques and factors influencing the properties of the encapsulating agents can be found in recent reviews [122,125,126,129,131].

The physical barrier provided by some compounds can be enhanced by adding additional aids. Electrostatic attraction and chemical bonding can be enhanced by reducing the distance between an extracellular protective agent and cells, thereby increasing survival [132]. In recent years, Maillard reaction products of protein–polysaccharide have received more attention because Maillard reaction has the potential to enhance the encapsulation by promoting the covalent bond between carbolyl compounds and the amino group of a protein [133]. For example, whey protein isolate and dextran [134] or soy protein isolate and sodium carboxymethyl cellulose [135] lead to a higher bacterial survival rate after spray drying. Another example is the addition of calcium when proteins are used to coat the microorganisms, since it can induce protein aggregation [136,137]. Heat treatment of skim milk with added calcium causes milk proteins to aggregate. Cell survival appears to be correlated with the degrees of protein aggregation, as observed by Huang et al. [136]. Transglutaminase, a food enzyme, is also used to increase coating/encapsulation by inducing protein aggregation. Liu et al. [138] observed that structural modifications of soybean protein isolate by transglutaminase conferred an enhanced heat resistance to several lactic acid bacteria strains. Xiao et al. [139] reported similar results for two strains of lactobacillus encapsulated by whey protein isolate cross-linked by transglutaminase. However, they found that the protective effect of this encapsulation was not satisfactory for freeze-drying. This barrier effect provided by these compounds can have an impact on drying kinetics. In fact, the addition of high molecular weight compounds to the surface of the product can lead to an increase in size and therefore drying time, which can be harmful to the cells [124]. In addition, a slower rate of temperature increase due to the crust can be observed and thus less stress upon the cells [119]. The change in drying kinetics and solid temperature is a mechanism that can be beneficial or detrimental to the survival of microorganisms and is discussed in the Section 2.7. These protective agents can slow the arrival of heat into the cell.

### 2.6. Thermal Resistance Enhancement

Some compounds could improve the thermal resistance of microorganisms, for example by improving protein stability [5] or by stimulating the cells’ own protective mechanisms. For example, Wang et al. [140] observed that adding calcium to the growth medium could help to increase the activity of the heat shock protein, thereby enhancing protection during stress. In our view, this mechanism serves as a broad framework, often employed when deeper research and greater understanding are needed. We include it to account for processes discussed in the literature that have not been explicitly presented here, but for which the specifics of the thermal resistance improvement remain challenging to describe.

### 2.7. Change in Drying Kinetics or Product Temperature

**Figure 4:**
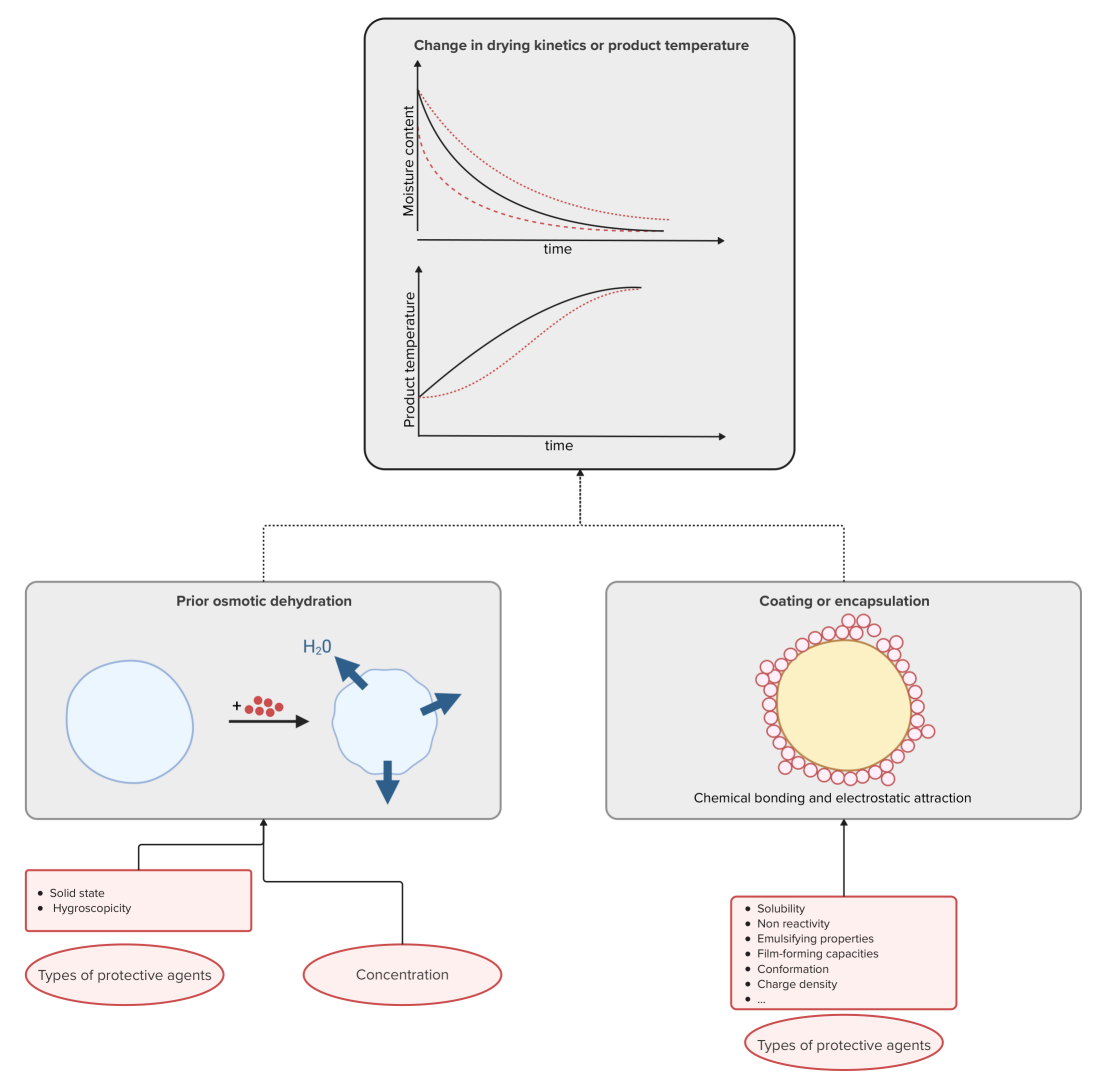
Diagram summarizing the mechanism of coating or encapsulation, prior osmotic dehydration, change in drying kinetics and their possible interactions. The figure contains graphics created in BioRender. Van Engeland, C. (2025) https://BioRender.com/s09w969/ (accessed on 3 February 2025). The red symbols in the graphical illustrations of the mechanisms represent a protective agent. The oval boxes represent operating conditions that can be modified.

As discussed in Section 2.3 and Section 2.5, the addition of protective agents can, in some cases, lead to a change in drying kinetics that can affect the viability of microorganisms. Figure 4 highlights that, in some cases, coating and osmotic dehydration can lead to a change in the drying kinetics and temperature evolution of the product under study. Figure 4 also provides a schematic illustration of these mechanisms. The effect of drying kinetics, residual moisture content and product temperature evolution on microbial viability has been demonstrated in several studies [6,12,141,142,143]. The addition of protective compounds has the potential to alter these variables, which may subsequently affect the viability of the product. In fact, the viability of microorganisms is closely linked to their water content. Several researchers have shown that at the end of the drying process, viability decreases abruptly as the water content decreases [12,106,120,137]. Thus, changing the residual moisture content by adding a protective agent will also change the final quality of the product. Regarding the kinetics, slowing down the moisture removal could have a positive effect on the final viability [142,143]. However, the longer the drying time, the longer the microorganisms are exposed to stress and this can lead to loss of viability [3,12]. Indeed, slow drying times result in a slower decrease in water activity, but they also lead to an increase in dehydration inactivation due to a longer residence time in a critical range of water activity [12,144,145]. As a result, an optimal kinetics can be determined [144,145]. The change in particle size after coating can be very substantial and therefore lead to a significant change in drying kinetics. For example, Chandralekha et al. [146] observed dry particles ranging from 10 µm without any carrier material to about 400 µm for the highest particle size with carrier during spray drying of yeast. Cell survival was improved by using protective agents that moderate the drying rate so that a low moisture content can be achieved at a lower temperature [50]. Khem et al. [119] argued that the rate of temperature change is a key factor contributing to cell death and that reducing the rate of temperature increase leads to a better protection. Indeed, it appears that the combination of high temperature and high humidity is detrimental to cells. Better preservation may therefore be achieved by reducing the rate of temperature increase. Modification of drying kinetics is not the only key factor related to cell survival. Indeed, Liu et al. [21] observed that *Lactobacillus rhamnosus GG* with trehalose or lactose had comparable water removal and temperature profiles during drying. However, trehalose appeared to offer a superior protective effect compared with lactose.

## 3. Discussion

In this review, seven main mechanisms have been brought to light, most of which attempt to mimic or enhance the adaptive responses of probiotics to various stresses. These mechanisms may result in protection during the drying phase and/or during prolonged storage of the microorganisms. These mechanisms have been suggested in the literature but to our knowledge, no review has attempted to address them all. In fact, the most recent reviews on the subject focus mainly on the different molecules that can be used and generally for a specific drying technique [10,37,61,147]. However, depending on the drying technique, the stresses experienced by the cells will be different and therefore the protective mechanisms to be promoted may also be different.

**Figure 5:**
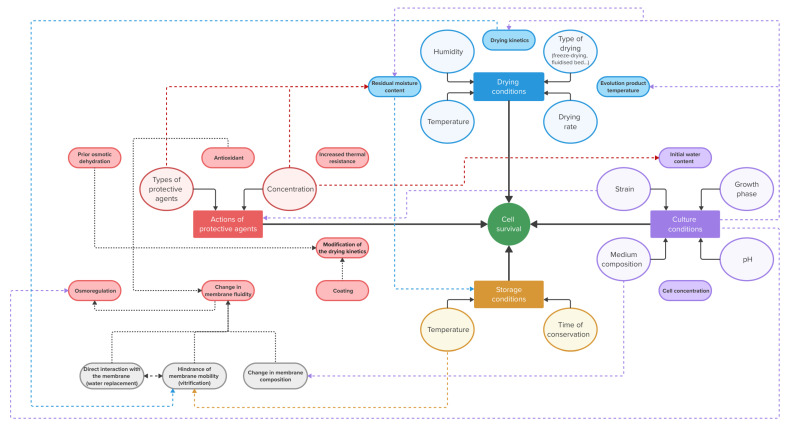
Diagram summarizing the relations between various conditions affecting cell survival, as well as the relations between the different protection mechanisms of protective agents. The color scheme used in the figure is as follows: red corresponds to the mechanisms of action of protective agents, purple refers to the culture conditions, blue corresponds to the drying conditions, and yellow refers to the storage conditions. The circular boxes represent operating conditions that can be subject to modification.

In addition, given the large number of compounds that can be tested, it would be more relevant to start from the mechanisms that are being targeted and see which protective agents can be considered. Nevertheless, as seen in Section 2, some mechanisms are closely related. The link between the different mechanisms and their influence on different parameters that can affect cell survival is illustrated in Figure 5. This figure illustrates the difficulty of estimating cell survival and understanding protection mechanisms because many interdependent variables can influence survival.

Depending on the physicochemical properties of the protective agents, different mechanisms can be used to protect the microorganisms. As discussed in Section 2, the relevance of a mechanism is contingent upon the size, hygroscopicity and chemical properties of the protective agent, including its capacity to gel, glass-transition temperature and functional groups, among other factors. A number of compounds can induce more than one protective mechanism. The best known example is trehalose, which is widely known to enhance the ability of cells to tolerate desiccation [34,148]. In addition to reducing lipid peroxidation [112], trehalose is thought to affect membrane fluidity by replacing water molecules and vitrifying [34]. Reconstituted skimmed milk (RSM) has also been identified as a promising protective agent due to its composition, which includes a number of potential protective agents, such as various proteins and sugars. Drying kinetics and temperature variation during drying can be influenced by the coating of cells by RSM [119,120]. RSM, which contains Ca^2+^, has the ability to combine with proteins to form agglomerates that can subsequently coat cells as shown by Huang et al. [136]. Furthermore, it has been suggested that lactose in RSM may also help to maintain membrane integrity [149]. These results show that the incorporation of RSM may have a synergistic effect by combining three different mechanisms—coating, modification of drying kinetics and membrane fluidity. However, RSM is a known allergen, and therefore its use is not appropriate in all circumstances. Therefore, other combinations of compounds are being investigated. In recent years, a number of studies have shown that a combination of protective agents can provide better protection of microorganisms than these compounds alone [17,18,83,150,151,152]. For example, How et al. [152] observed a synergistic effect of maltodextrine and trehalose as a lyoprotectant. This mixture resulted in an increase in viability and a decrease in moisture content. Chin et al. [18] have found that a combination of skim milk, maltose and maltitol can result in a high survival rate during freeze-drying. It is likely that the skim milk acts as a coating mechanism, that the storage effect is improved by an antioxidant and finally that the low sugar has an effect on the stability of the membrane. In addition, the addition of a complex protective agent could also increase the content of unsaturated fatty acids in the cell membrane. Cheng et al. [17] observed that the unsaturated fatty acid content—which appears to improve the viability of the product after drying (see Section 2.1.3)—increased more with a combination of potential protective agents than with the compounds alone. Finally, some protective agents may have a positive effect on drying but may not provide any protection during storage and vice versa [56,153]. Therefore, a combination of protective agents could procure a protection over the whole process, drying, storage and rehydration. The aforementioned research findings highlight that a synergistic effect can be achieved through the combined use of different protective agents. It is therefore relevant and necessary to be able to determine the protective role they may have in order to combine them in the best possible way.

In some conditions, the incorporation of protective agents can have a negative impact on cell quality. For example, Rodklongtan et al. [112] showed that ascorbic acid at 24 mg/mL had a negative effect on viability but promoted survival at lower concentrations. The authors suggested that this negative effect was the result of an important change in fluidity, as evidenced by a significant decrease in $T_{g}$ upon the addition of ascorbic acid. Ascorbic acid thus has a dual effect: at low concentrations it helps to reduce oxidation, but at high concentrations it increases molecular mobility, which can promote cell death during storage. Another way that leads to a decrease in viability is an osmotic shock due to the addition of a significant concentration of protective agents. A previous research by Van Engeland et al. [106] highlights that the addition of flour to yeast paste leads to a decrease of fermentation activity probably due to osmotic shock. The complexity lies in the fact that several mechanisms may act simultaneously, some of which may be beneficial to the cell, while others may be detrimental.

It is worth underlining the importance of adapting the potential protective agents according to the size of the product, the drying technique and also the strain studied. A number of studies have shown that the effects of different stress and cryoprotectants may vary depending on the strain [154,155,156]. For example, Khem et al. [124] have shown that two strains of *L. plantarum* with different levels of hydrophobicity require different concentrations of protective medium to embed the cells and enhance the viability. Stefanello et al. [155] observed that trehalose conferred a more effective protective effect on yeast cells of Wickerhamomyces than on bacterial cells of lactobacillus fermentum. Zemancikova et al. [156] show that, despite the similarity of the physiological characteristics of four species, they exhibit different tolerances to hydrobiosis and osmotolerance. They propose that this discrepancy is due to their ability to metabolize in a fluctuating environment. One of the challenges in this area of research is that different species have different levels of tolerance to different stresses. Consequently, a mechanism that may be beneficial to one strain may have no effect or even be detrimental to another.

It is important to note that comparisons between the various studies are not straightforward due to the use of different strains, different drying techniques and different concentrations of protective compounds. Further research is needed to better understand this issue, as comparative analyzes may help to develop guidelines to improve microbial viability during drying and storage. As illustrated in Figure 5, a comparative analysis of the various studies is challenging due to the multitude of factors that affect cell survival [157]. These factors vary across studies and include drying techniques, growth conditions, storage conditions, cell strains used and other variables. Nevertheless, there are some points that may facilitate the comparison of studies and thus improve our understanding of the underlying mechanisms. For example, several researchers have shown that the quality of the microorganisms strongly depends on the moisture content [1,11,12,143]. The addition of a protective agent could affect the residual moisture content [83] and, consequently, the final quality. It is therefore advisable to always compare the viability and residual moisture content. However, the residual moisture content is not always mentioned, which makes the comparison difficult. To ease the comparison, it would be interesting to always have information on the initial and residual moisture content. Therefore, analyzing the evolution of the viability during drying seems to be an interesting point of view to try to improve the understanding of the mechanisms taking place during drying. Since the quality of the microorganisms also depends on the drying kinetics (see Section 2.7), this effect should not be overlooked. It seems essential to determine whether the addition has an effect on external factors, internal factors or both. It may be interesting to conduct a robust study of some key preservatives that we believe have only one predominant protective mechanism. This study would include a thorough analysis of the state of the membrane before and after drying (fluidity, glass-transition temperature, membrane phase-transition temperature, composition), intracellular content, osmotic shock estimation and drying kinetics.

## 4. Conclusions

This review provides a broad and integrative perspective of the mechanisms through which protective agents may act during the drying of microorganisms. It outlines seven potential mechanisms: (i) change of membrane fluidity, (ii) osmoregulation, (iii) prior osmotic dehydration, (iv) oxidation prevention, (v) coating or encapsulation, (vi) enhanced thermal resistance and (vii) change in drying kinetics. These mechanisms, several of which seek to mimic the adaptive responses of cells to various stresses, result in internal or external modifications of the cells. Figure 5 provides a graphical summary of this review, illustrating the mechanisms, their interconnections and the influence of the operating conditions, the strains and protective agents involved. The figure shows that the survival of microorganisms is influenced by four primary factors: the drying conditions, the storage conditions, the culture conditions and the action of protective agents. It also highlights the complexity involved in studying protection mechanisms. The review emphasizes the necessity for fine-tuning to enhance survival, as excessive modifications can be detrimental to the cells. Indeed, multiple mechanisms may act simultaneously, with some being beneficial to the cells while others may be detrimental. Protection mechanisms can also mutually influence each other and are contingent upon the operating conditions and the physicochemical properties of the protective agents involved. This review also highlights that a deeper understanding of these mechanisms is needed to help define strategies to increase the quality of the product obtained after drying, for example by facilitating the combination of several agents to obtain a synergistic effect. To improve the survival of microorganisms through drying and storage, further in-depth comparative analyses across different conditions are needed.

## Abbreviations

The following abbreviations are used in this manuscript:

CFA	Cyclic fatty acid
ROS	Reactive oxygen species
RSM	Reconstituted skimmed milk
SFA	Saturated fatty acid
$T_{m}$	Membrane phase-transition temperature
$T_{g}$	Glass-transition temperature
UFA	Unsaturated fatty acid

## References

[1] Bayrock D., Ingledew W.M. (1997). Mechanism of viability loss during fluidized bed drying of baker’s yeast. Food Res. Int..

[2] Lemetais G., Dupont S., Beney L., Gervais P. (2012). Air-drying kinetics affect yeast membrane organization and survival. Appl. Microbiol. Biotechnol..

[3] Fu N., Chen X.D. (2011). Towards a maximal cell survival in convective thermal drying processes. Food Res. Int..

[4] Sehrawat R., Abdullah S., Khatri P., Kumar L., Kumar A., Mujumdar A.S. (2022). Role of drying technology in probiotic encapsulation and impact on food safety. Dry. Technol..

[5] Santivarangkna C., Kulozik U., Foerst P. (2008). Inactivation Mechanisms of Lactic Acid Starter Cultures Preserved by Drying Processes. J. Appl. Microbiol..

[6] Mille Y., Girard J., Beney L., Gervais P. (2005). Air drying optimization of Saccharomyces cerevisiae through its water–glycerol dehydration properties. J. Appl. Microbiol..

[7] Garre E., Raginel F., Palacios A., Julien A., Matallana E. (2010). Oxidative stress responses and lipid peroxidation damage are induced during dehydration in the production of dry active wine yeasts. Int. J. Food Microbiol..

[8] Somero G.N. (1995). Proteins and Temperature. Annu. Rev. Physiol..

[9] Laroche C., Beney L., Marechal P., Gervais P. (2001). The Effect of Osmotic Pressure on the Membrane Fluidity of Saccharomyces Cerevisiae at Different Physiological Temperatures. Appl. Microbiol. Biotechnol..

[10] Wang N., Fu N., Chen X.D. (2022). The extent and mechanism of the effect of protectant material in the production of active lactic acid bacteria powder using spray drying: A review. Curr. Opin. Food Sci..

[11] Fu N., Woo M.W., Selomulya C., Chen X.D. (2013). Inactivation of *Lactococcus lactis* ssp. *cremoris* cells in a droplet during convective drying. Biochem. Eng. J..

[12] Perdana J., Bereschenko L., Fox M.B., Kuperus J.H., Kleerebezem M., Boom R.M., Schutyser M.A.I. (2013). Dehydration and thermal inactivation of Lactobacillus plantarum WCFS1: Comparing single droplet drying to spray and freeze drying. Food Res. Int..

[13] Leuenberger P., Ganscha S., Kahraman A., Cappelletti V., Boersema P.J., von Mering C., Claassen M., Picotti P. (2017). Cell-wide analysis of protein thermal unfolding reveals determinants of thermostability. Science.

[14] Ge S., Han J., Sun Q., Zhou Q., Ye Z., Li P., Gu Q. (2024). Research progress on improving the freeze-drying resistance of probiotics: A review. Trends Food Sci. Technol..

[15] Asioli D., Aschemann-Witzel J., Caputo V., Vecchio R., Annunziata A., Næs T., Varela P. (2017). Making sense of the “clean label” trends: A review of consumer food choice behavior and discussion of industry implications. Food Res. Int..

[16] Vargas M.C.A., Simsek S. (2021). Clean Label in Bread. Foods.

[17] Cheng Z., Yan X., Wu J., Weng P., Wu Z. (2022). Effects of freeze drying in complex lyoprotectants on the survival, and membrane fatty acid composition of Lactobacillus plantarum L1 and Lactobacillus fermentum L2. Cryobiology.

[18] Chin Y.W., Lee S., Yu H.H., Yang S.J., Kim T.W. (2021). Combinatorial Effects of Protective Agents on Survival Rate of the Yeast Starter, Saccharomyces cerevisiae 88-4, after Freeze-Drying. Microorganisms.

[19] Gaucher F., Rabah H., Kponouglo K., Bonnassie S., Pottier S., Dolivet A., Marchand P., Jeantet R., Blanc P., Jan G. (2020). Intracellular osmoprotectant concentrations determine Propionibacterium freudenreichii survival during drying. Appl. Microbiol. Biotechnol..

[20] Gong P., Lin K., Zhang J., Han X., Lyu L., Yi H., Sun J., Zhang L. (2020). Enhancing spray drying tolerance of Lactobacillus bulgaricus by intracellular trehalose delivery via electroporation. Food Res. Int..

[21] Liu B., Fu N., Woo M.W., Chen X.D. (2018). Heat stability of Lactobacillus rhamnosus GG and its cellular membrane during droplet drying and heat treatment. Food Res. Int..

[22] Rockinger U., Funk M., Winter G. (2021). Current approaches of preservation of cells during (freeze-) drying. J. Pharm. Sci..

[23] Billi D., Potts M. (2002). Life and death of dried prokaryotes. Res. Microbiol..

[24] Kathiriya M.R., Vekariya Y.V., Hati S. (2023). Understanding the Probiotic Bacterial Responses Against Various Stresses in Food Matrix and Gastrointestinal Tract: A Review. Probiotics Antimicrob. Proteins.

[25] Gao X., Kong J., Zhu H., Mao B., Cui S., Zhao J. (2022). Lactobacillus, Bifidobacterium and Lactococcus response to environmental stress: Mechanisms and application of cross-protection to improve resistance against freeze-drying. J. Appl. Microbiol..

[26] Gaucher F., Bonnassie S., Rabah H., Blanc P., Jeantet R., Jan G. (2019). Review: Adaptation of Beneficial Propionibacteria, Lactobacilli, and Bifidobacteria Improves Tolerance Toward Technological and Digestive Stresses. Front. Microbiol..

[27] Chávez B., Ledeboer A. (2007). Drying of probiotics: Optimization of formulation and process to enhance storage survival. Dry. Technol..

[28] Moayyedi M., Eskandari M.H., Rad A.H.E., Ziaee E., Khodaparast M.H.H., Golmakani M.T. (2018). Effect of drying methods (electrospraying, freeze drying and spray drying) on survival and viability of microencapsulated Lactobacillus rhamnosus ATCC 7469. J. Funct. Foods.

[29] Meneghel J., Passot S., Cenard S., Réfrégiers M., Jamme F., Fonseca F. (2017). Subcellular membrane fluidity of Lactobacillus delbrueckii subsp. bulgaricus under cold and osmotic stress. Appl. Microbiol. Biotechnol..

[30] Simonin H., Beney L., Gervais P. (2008). Controlling the membrane fluidity of yeasts during coupled thermal and osmotic treatments. Biotechnol. Bioeng..

[31] Guyot S., Ferret E., Gervais P. (2006). Yeast Survival during Thermal and Osmotic Shocks Is Related to Membrane Phase Change. J. Agric. Food Chem..

[32] Alberts B., Johnson A., Lewis J., Raff M., Roberts K., Walter P. (2002). The Lipid Bilayer. Molecular Biology of the Cell.

[33] Wolfe J., Bryant G. (1999). Freezing, Drying, and/or Vitrification of Membrane– Solute–Water Systems. Cryobiology.

[34] Leslie S.B., Teter S.A., Crowe L.M., Crowe J.H. (1994). Trehalose lowers membrane phase transitions in dry yeast cells. Biochim. Biophys. Acta (BBA)-Biomembr..

[35] Laroche C., Gervais P. (2003). Achievement of rapid osmotic dehydration at specific temperatures could maintain high Saccharomyces cerevisiae viability. Appl. Microbiol. Biotechnol..

[36] Crowe J.H., Crowe L.M., Chapman D. (1984). Preservation of Membranes in Anhydrobiotic Organisms: The Role of Trehalose. Science.

[37] Santivarangkna C., Higl B., Foerst P. (2008). Protection mechanisms of sugars during different stages of preparation process of dried lactic acid starter cultures. Food Microbiol..

[38] Crowe J.H., Crowe L.M., Carpenter J.F., Aurell Wistrom C. (1987). Stabilization of dry phospholipid bilayers and proteins by sugars. Biochem. J..

[39] Crowe J.H., Crowe L.M., Carpenter J.F., Rudolph A.S., Wistrom C.A., Spargo B.J., Anchordoguy T.J. (1988). Interactions of sugars with membranes. Biochim. Biophys. Acta (BBA)-Rev. Biomembr..

[40] Lee C.W.B., Das Gupta S.K., Mattai J., Shipley G.G., Abdel-Mageed O.H., Makriyannis A., Griffin R.G. (1989). Characterization of the L.lambda. phase in trehalose-stabilized dry membranes by solid-state NMR and x-ray diffraction. Biochemistry.

[41] Sum A.K., Faller R., de Pablo J.J. (2003). Molecular Simulation Study of Phospholipid Bilayers and Insights of the Interactions with Disaccharides. Biophys. J..

[42] Vereyken I.J., Chupin V., Hoekstra F.A., Smeekens S.C., De Kruijff B. (2003). The Effect of Fructan on Membrane Lipid Organization and Dynamics in the Dry State. Biophys. J..

[43] Oldenhof H., Wolkers W.F., Fonseca F., Passot S., Marin M. (2005). Effect of Sucrose and Maltodextrin on the Physical Properties and Survival of Air-Dried Lactobacillus bulgaricus: An in Situ Fourier Transform Infrared Spectroscopy Study. Biotechnol. Prog..

[44] Tonnis W.F., Mensink M.A., de Jager A., van der Voort Maarschalk K., Frijlink H.W., Hinrichs W.L.J. (2015). Size and Molecular Flexibility of Sugars Determine the Storage Stability of Freeze-Dried Proteins. Mol. Pharm..

[45] Pereira C.S., Lins R.D., Chandrasekhar I., Freitas L.C.G., Hünenberger P.H. (2004). Interaction of the disaccharide trehalose with a phospholipid bilayer: A molecular dynamics study. Biophys. J..

[46] Disalvo E.A., Lairion F., Martini F., Tymczyszyn E., Frías M., Almaleck H., Gordillo G.J. (2008). Structural and functional properties of hydration and confined water in membrane interfaces. Biochim. Biophys. Acta (BBA)-Biomembr..

[47] Luzardo M.d.C., Amalfa F., Nunez A., Diaz S., De Lopez A.B., Disalvo E. (2000). Effect of trehalose and sucrose on the hydration and dipole potential of lipid bilayers. Biophys. J..

[48] Bryant G., Koster K.L., Wolfe J. (2001). Membrane behaviour in seeds and other systems at low water content: The various effects of solutes. Seed Sci. Res..

[49] Kumara U.G.V.S.S., Ramirez J.F., Boothby T.C. (2024). The effect of sucrose polymer-size on glass transition temperature, glass former fragility, and water retention during drying. Front. Mater..

[50] Ghandi A., Powell I.B., Chen X.D., Adhikari B. (2013). The Survival of Lactococcus lactis in a Convective-Air-Drying Environment: The Role of Protectant Solids, Oxygen Injury, and Mechanism of Protection. Dry. Technol..

[51] Tantratian S., Pradeamchai M. (2020). Select a protective agent for encapsulation of Lactobacillus plantarum. LWT.

[52] Siemons I., Vaessen E.M.J., Oosterbaan van Peski S.E., Boom R.M., Schutyser M.A.I. (2021). Protective effect of carrier matrices on survival of Lactobacillus plantarum WCFS1 during single droplet drying explained by particle morphology development. J. Food Eng..

[53] Perdana J., Fox M.B., Siwei C., Boom R.M., Schutyser M.A. (2014). Interactions between formulation and spray drying conditions related to survival of Lactobacillus plantarum WCFS1. Food Res. Int..

[54] Umashankar K., Chandralekha A., Dandavate T., Tavanandi H.A., Raghavarao K.S.M.S. (2019). A nonconventional method for drying of Pseudomonas aeruginosa and its comparison with conventional methods. Dry. Technol..

[55] Lee K., Koyama K., Kawai K., Koseki S. (2021). Why Does Cronobacter sakazakii Survive for a Long Time in Dry Environments? Contribution of the Glass Transition of Dried Bacterial Cells. Microbiol. Spectr..

[56] Romano N., Schebor C., Mobili P., Gómez-Zavaglia A. (2016). Role of mono- and oligosaccharides from FOS as stabilizing agents during freeze-drying and storage of Lactobacillus delbrueckii subsp. bulgaricus. Food Res. Int..

[57] Guerrero Sanchez M., Passot S., Ghorbal S., Campoy S., Olivares M., Fonseca F. (2023). Insights into the mechanisms of L. salivarius CECT5713 resistance to freeze-dried storage. Cryobiology.

[58] Gagneten M., Passot S., Cenard S., Ghorbal S., Schebor C., Fonseca F. (2024). Mechanistic study of the differences in lactic acid bacteria resistance to freeze- or spray-drying and storage. Appl. Microbiol. Biotechnol..

[59] Kurtmann L., Carlsen C.U., Risbo J., Skibsted L.H. (2009). Storage stability of freeze–dried *Lactobacillus acidophilus* (La-5) in relation to water activity and presence of oxygen and ascorbate. Cryobiology.

[60] Mikajiri S., Sogabe T., Cao R., Kikawada T., Suzuki T., Kawai K. (2022). Glass transition behavior of carnosine and its impact as a protectant on freeze-dried lactic acid bacteria. Food Biophys..

[61] Mensink M.A., Frijlink H.W., van der Voort Maarschalk K., Hinrichs W.L.J. (2017). How sugars protect proteins in the solid state and during drying (review): Mechanisms of stabilization in relation to stress conditions. Eur. J. Pharm. Biopharm..

[62] Patist A., Zoerb H. (2005). Preservation mechanisms of trehalose in food and biosystems. Colloids Surf. Biointerfaces.

[63] Hincha D.K., Popova A.V., Cacela C., Liu A.L. (2006). Chapter 6–Effects of Sugars on the Stability and Structure of Lipid Membranes During Drying. Advances in Planar Lipid Bilayers and Liposomes.

[64] Deng L., Wang Y., Jiang H., Xu X., Han J., Liu W. (2023). Specific protection mechanism of oligosaccharides on liposomes during freeze-drying. Food Res. Int..

[65] Denich T.J., Beaudette L.A., Lee H., Trevors J.T. (2003). Effect of selected environmental and physico-chemical factors on bacterial cytoplasmic membranes. J. Microbiol. Methods.

[66] Huang R., Pan M., Wan C., Shah N.P., Tao X., Wei H. (2016). Physiological and transcriptional responses and cross protection of Lactobacillus plantarum ZDY2013 under acid stress. J. Dairy Sci..

[67] Haddaji N., Mahdhi A.K., Krifi B., Ismail M.B., Bakhrouf A. (2015). Change in cell surface properties of Lactobacillus casei under heat shock treatment. FEMS Microbiol. Lett..

[68] Wang Y., Delettre J., Guillot A., Corrieu G., Béal C. (2005). Influence of cooling temperature and duration on cold adaptation of Lactobacillus acidophilus RD758. Cryobiology.

[69] Moorman M.A., Thelemann C.A., Zhou S., Pestka J.J., Linz J.E., Ryser E.T. (2008). Altered Hydrophobicity and Membrane Composition in Stress-Adapted Listeria innocua. J. Food Prot..

[70] Yang X., Hang X., Zhang M., Liu X., Yang H. (2015). Relationship between acid tolerance and cell membrane in Bifidobacterium, revealed by comparative analysis of acid-resistant derivatives and their parental strains grown in medium with and without Tween 80. Appl. Microbiol. Biotechnol..

[71] Beney L., Gervais P. (2001). Influence of the fluidity of the membrane on the response of microorganisms to environmental stresses. Appl. Microbiol. Biotechnol..

[72] Fonseca F., Pénicaud C., Tymczyszyn E.E., Gómez-Zavaglia A., Passot S. (2019). Factors influencing the membrane fluidity and the impact on production of lactic acid bacteria starters. Appl. Microbiol. Biotechnol..

[73] Marsh D. (2013). Handbook of Lipid Bilayers.

[74] Renne M.F., de Kroon A.I.P.M. (2018). The role of phospholipid molecular species in determining the physical properties of yeast membranes. FEBS Lett..

[75] Fábián B., Vattulainen I., Javanainen M. (2023). Protein Crowding and Cholesterol Increase Cell Membrane Viscosity in a Temperature Dependent Manner. J. Chem. Theory Comput..

[76] Louesdon S., Charlot-Rougé S., Juillard V., Tourdot-Maréchal R., Béal C. (2014). Osmotic stress affects the stability of freeze-dried Lactobacillus buchneri R1102 as a result of intracellular betaine accumulation and membrane characteristics. J. Appl. Microbiol..

[77] Cui S., Hu K., Qian Z., Mao B., Zhang Q., Zhao J., Tang X., Zhang H. (2022). Improvement of Freeze-Dried Survival of Lactiplantibacillus plantarum Based on Cell Membrane Regulation. Microorganisms.

[78] Jingjing E., Lili M., Zichao C., Rongze M., Qiaoling Z., Ruiyin S., Zongbai H., Junguo W. (2020). Effects of buffer salts on the freeze-drying survival rate of *Lactobacillus plantarum* LIP-1 based on transcriptome and proteome analyses. Food Chem..

[79] Chen Z., E J., Ma R., Zhang J., Yao C., Wang R., Zhang Q., Yang Y., Li J., Wang J. (2022). The effect of aspartic acid on the freeze-drying survival rate of Lactobacillus plantarum LIP-1 and its inherent mechanism. LWT.

[80] Wang G., Chen P., Yu X., Xia Y., Yan L.T., Ai L. (2020). C18:1 Improves the Freeze-Drying Resistance of Lactobacillus plantarum by Maintaining the Cell Membrane. ACS Appl. Bio Mater..

[81] Wang G., Pu J., Dong C., Zheng X., Guo B., Xia Y., Ai L. (2021). Effect of oleic acid on the viability of different freeze-dried *Lactiplantibacillus plantarum* strains. J. Dairy Sci..

[82] Li B., Tian F., Liu X., Zhao J., Zhang H., Chen W. (2011). Effects of cryoprotectants on viability of Lactobacillus reuteri CICC6226. Appl. Microbiol. Biotechnol..

[83] Li X.M., Che L.H., Wu Y., Li C., Xu B.C. (2024). An effective strategy for improving the freeze-drying survival rate of *Lactobacillus curvatus* and its potential protective mechanism. Food Biosci..

[84] Sompach G., Rodklongtan A., Nitisinprasert S., Chitprasert P. (2022). Microencapsulating role of whey protein isolate and sucrose in protecting the cell membrane and enhancing survival of probiotic lactobacilli strains during spray drying, storage, and simulated gastrointestinal passage. Food Res. Int..

[85] Velly H., Bouix M., Passot S., Penicaud C., Beinsteiner H., Ghorbal S., Lieben P., Fonseca F. (2015). Cyclopropanation of unsaturated fatty acids and membrane rigidification improve the freeze-drying resistance of Lactococcus lactis subsp. lactis TOMSC161. Appl. Microbiol. Biotechnol..

[86] Guillot A., Obis D., Mistou M.Y. (2000). Fatty acid membrane composition and activation of glycine-betaine transport in *Lactococcus lactis* subjected to osmotic stress. Int. J. Food Microbiol..

[87] Girardeau A., Passot S., Meneghel J., Cenard S., Lieben P., Trelea I.C., Fonseca F. (2022). Insights into lactic acid bacteria cryoresistance using FTIR microspectroscopy. Anal. Bioanal. Chem..

[88] Arora A., Raghuraman H., Chattopadhyay A. (2004). Influence of cholesterol and ergosterol on membrane dynamics: A fluorescence approach. Biochem. Biophys. Res. Commun..

[89] Bonaterra A., Camps J., Montesinos E. (2005). Osmotically induced trehalose and glycine betaine accumulation improves tolerance to desiccation, survival and efficacy of the postharvest biocontrol agent Pantoea agglomerans EPS125. FEMS Microbiol. Lett..

[90] Cerrutti P., Segovia de Huergo M., Galvagno M., Schebor C., del Pilar Buera M. (2000). Commercial baker’s yeast stability as affected by intracellular content of trehalose, dehydration procedure and the physical properties of external matrices. Appl. Microbiol. Biotechnol..

[91] Cui S., Zhou W., Tang X., Zhang Q., Yang B., Zhao J., Mao B., Zhang H. (2022). The Effect of Proline on the Freeze-Drying Survival Rate of Bifidobacterium longum CCFM 1029 and Its Inherent Mechanism. Int. J. Mol. Sci..

[92] Eardley J., Timson D.J. (2020). Yeast cellular stress: Impacts on bioethanol production. Fermentation.

[93] Hohmann S. (2002). Osmotic stress signaling and osmoadaptation in yeasts. Microbiol. Mol. Biol. Rev..

[94] Vaessen E.M.J., den Besten H.M.W., Esveld E.D.C., Schutyser M.A.I. (2019). Accumulation of intracellular trehalose and lactose in *Lactobacillus plantarum* WCFS1 during pulsed electric field treatment and subsequent freeze and spray drying. LWT.

[95] Linders L., Meerdink G., Van ‘t Riet K. (1997). Effect of growth parameters on the residual activity of Lactobacillus plantarum after drying. J. Appl. Microbiol..

[96] Kets E.P., De Bont J.A. (1994). Protective effect of betaine on survival of Lactobacillus plantarum subjected to drying. FEMS Microbiol. Lett..

[97] Martos G., Minahk C., Font de Valdez G., Morero R. (2007). Effects of protective agents on membrane fluidity of freeze-dried Lactobacillus delbrueckii ssp. bulgaricus. Lett. Appl. Microbiol..

[98] Louis P., Trüper H., Galinski E. (1994). Survival of Escherichia coli during drying and storage in the presence of compatible solutes. Appl. Microbiol. Biotechnol..

[99] van der Heide T., Poolman B. (2000). Osmoregulated ABC-transport system of Lactococcus lactis senses water stress via changes in the physical state of the membrane. Proc. Natl. Acad. Sci. USA.

[100] Prasad J., McJarrow P., Gopal P. (2003). Heat and Osmotic Stress Responses of Probiotic Lactobacillus rhamnosus HN001 (DR20) in Relation to Viability after Drying. Appl. Environ. Microbiol..

[101] Ramya V., Jain N.K. (2017). A Review on Osmotic Dehydration of Fruits and Vegetables: An Integrated Approach. J. Food Process. Eng..

[102] Simonin H., Beney L., Gervais P. (2007). Sequence of occurring damages in yeast plasma membrane during dehydration and rehydration: Mechanisms of cell death. Biochim. Biophys. Acta (BBA)-Biomembr..

[103] Mille Y., Obert J.P., Beney L., Gervais P. (2004). New drying process for lactic bacteria based on their dehydration behavior in liquid medium. Biotechnol. Bioeng..

[104] Laroche C., Gervais P. (2003). Unexpected Thermal Destruction of Dried, Glass Bead-Immobilized Microorganisms as a Function of Water Activity. Appl. Environ. Microbiol..

[105] Liu X.D., Zbicinski I., Strumillo C. (2003). Quality Enhancement for Thermosensitive Materials Dried with Solid Carriers. Dry. Technol..

[106] Van Engeland C., Aujard M., Rabineau J., Spreutels L., Legros R., Haut B. (2022). Evolution of the quality of baker’s yeast pellets containing a carrier during their convective drying. Dry. Technol..

[107] Laroche C., Fine F., Gervais P. (2005). Water activity affects heat resistance of microorganisms in food powders. Int. J. Food Microbiol..

[108] Len J.S., Koh W.S.D., Tan S.X. (2019). The roles of reactive oxygen species and antioxidants in cryopreservation. Biosci. Rep..

[109] Halliwell B. (2006). Reactive Species and Antioxidants. Redox Biology Is a Fundamental Theme of Aerobic Life. Plant Physiol..

[110] França M.B., Panek A.D., Eleutherio E.C.A. (2007). Oxidative stress and its effects during dehydration. Comp. Biochem. Physiol. Part A Mol. Integr. Physiol..

[111] de Jesus Pereira E., Panek A.D., Eleutherio E.C.A. (2003). Protection against oxidation during dehydration of yeast. Cell Stress Chaperones.

[112] Rodklongtan A., Nitisinprasert S., Chitprasert P. (2022). Antioxidant activity and the survival-enhancing effect of ascorbic acid on Limosilactobacillus reuteri KUB-AC5 microencapsulated with lactose by spray drying. LWT.

[113] Dupont S., Fleurat-Lessard P., Cruz R.G., Lafarge C., Grangeteau C., Yahou F., Gerbeau-Pissot P., Abrahão Júnior O., Gervais P., Simon-Plas F. (2021). Antioxidant Properties of Ergosterol and Its Role in Yeast Resistance to Oxidation. Antioxidants.

[114] Herdeiro R.S., Pereira M.D., Panek A.D., Eleutherio E.C.A. (2006). Trehalose protects *Saccharomyces cerevisiae* from lipid peroxidation during oxidative stress. Biochim. Biophys. Acta (BBA)-Gen. Subj..

[115] Temple M.D., Perrone G.G., Dawes I.W. (2005). Complex cellular responses to reactive oxygen species. Trends Cell Biol..

[116] Borst J.W., Visser N.V., Kouptsova O., Visser A.J.W.G. (2000). Oxidation of unsaturated phospholipids in membrane bilayer mixtures is accompanied by membrane fluidity changes. Biochim. Biophys. Acta (BBA)-Mol. Cell Biol. Lipids.

[117] Teixeira P., Castro H., Kirby R. (1996). Evidence of membrane lipid oxidation of spray-dried Lactobacillus bulgaricus during storage. Lett. Appl. Microbiol..

[118] Oku K., Watanabe H., Kubota M., Fukuda S., Kurimoto M., Tsujisaka Y., Komori M., Inoue Y., Sakurai M. (2003). NMR and quantum chemical study on the OH··· *π* and CH··· O interactions between trehalose and unsaturated fatty acids: Implication for the mechanism of antioxidant function of trehalose. J. Am. Chem. Soc..

[119] Khem S., Woo M.W., Small D.M., Chen X.D., May B.K. (2015). Agent selection and protective effects during single droplet drying of bacteria. Food Chem..

[120] Zheng X., Fu N., Duan M., Woo M.W., Selomulya C., Chen X.D. (2015). The mechanisms of the protective effects of reconstituted skim milk during convective droplet drying of lactic acid bacteria. Food Res. Int..

[121] Duongthingoc D., George P., Katopo L., Gorczyca E., Kasapis S. (2013). Effect of whey protein agglomeration on spray dried microcapsules containing *Saccharomyces boulardii*. Food Chem..

[122] Nezamdoost-Sani N., Khaledabad M.A., Amiri S., Phimolsiripol Y., Khaneghah A.M. (2024). A comprehensive review on the utilization of biopolymer hydrogels to encapsulate and protect probiotics in foods. Int. J. Biol. Macromol..

[123] Rajam R., Anandharamakrishnan C. (2015). Microencapsulation of Lactobacillus plantarum (MTCC 5422) with fructooligosaccharide as wall material by spray drying. LWT-Food Sci. Technol..

[124] Khem S., Small D.M., May B.K. (2016). The behaviour of whey protein isolate in protecting Lactobacillus plantarum. Food Chem..

[125] Le X.T., Rioux L.E., Turgeon S.L. (2017). Formation and functional properties of protein–polysaccharide electrostatic hydrogels in comparison to protein or polysaccharide hydrogels. Adv. Colloid Interface Sci..

[126] Xie A., Zhao S., Liu Z., Yue X., Shao J., Li M., Li Z. (2023). Polysaccharides, proteins, and their complex as microencapsulation carriers for delivery of probiotics: A review on carrier types and encapsulation techniques. Int. J. Biol. Macromol..

[127] Stephen A.M., Phillips G.O. (2006). Food Polysaccharides and Their Applications.

[128] Desai K.G.H., Jin Park H. (2005). Recent developments in microencapsulation of food ingredients. Dry. Technol..

[129] Rodrigues F., Cedran M., Bicas J., Sato H. (2020). Encapsulated probiotic cells: Relevant techniques, natural sources as encapsulating materials and food applications–A narrative review. Food Res. Int..

[130] Afzaal M., Saeed F., Ateeq H., Imran A., Yasmin I., Shahid A., Javed A., Shah Y.A., Islam F., Ofoedu C.E. (2022). Survivability of probiotics under hostile conditions as affected by prebiotic-based encapsulating materials. Int. J. Food Prop..

[131] Akbarbaglu Z., Peighambardoust S.H., Sarabandi K., Jafari S.M. (2021). Spray drying encapsulation of bioactive compounds within protein-based carriers; different options and applications. Food Chem..

[132] Gong P., Di W., Yi H., Sun J., Zhang L., Han X. (2019). Improved viability of spray-dried Lactobacillus bulgaricus sp1. 1 embedded in acidic-basic proteins treated with transglutaminase. Food Chem..

[133] Akhtar M., Ding R. (2017). Covalently cross-linked proteins & polysaccharides: Formation, characterisation and potential applications. Curr. Opin. Colloid Interface Sci..

[134] Loyeau P.A., Spotti M.J., Vanden Braber N.L., Rossi Y.E., Montenegro M.A., Vinderola G., Carrara C.R. (2018). Microencapsulation of *Bifidobacterium animalis* subsp. *lactis* INL1 using whey proteins and dextrans conjugates as wall materials. Food Hydrocoll..

[135] Yan S., Wei J., Huang Y., Li Y., Yuan F., Liu J. (2024). Production of microcapsules by Maillard reaction of soy protein isolate and sodium carboxymethyl cellulose for protection of *bifidobacterium lactis*. Colloids Surfaces Physicochem. Eng. Asp..

[136] Huang S., Yang Y., Fu N., Qin Q., Zhang L., Chen X.D. (2014). Calcium-Aggregated Milk: A Potential New Option for Improving the Viability of Lactic Acid Bacteria Under Heat Stress. Food Bioprocess Technol..

[137] Wang J., Huang S., Fu N., Jeantet R., Chen X.D. (2016). Thermal Aggregation of Calcium-Fortified Skim Milk Enhances Probiotic Protection during Convective Droplet Drying. J. Agric. Food Chem..

[138] Liu J., Xie H., Gao Y., Zhu Y., Zhao H., Zhang B. (2023). Soybean protein isolate treated with transglutaminase (TGase) enhances the heat tolerance of selected lactic acid bacteria strains to spray drying. Food Chem..

[139] Xiao Y., Han C., Yang H., Liu M., Meng X., Liu B. (2020). Layer (whey protein isolate) -by-layer (xanthan gum) microencapsulation enhances survivability of *L. bulgaricus* and *L. paracasei* under simulated gastrointestinal juice and thermal conditions. Int. J. Biol. Macromol..

[140] Wang Y., Hao F., Lu W., Suo X., Bellenger E., Fu N., Jeantet R., Chen X.D. (2020). Enhanced thermal stability of lactic acid bacteria during spray drying by intracellular accumulation of calcium. J. Food Eng..

[141] Chen X.D., Patel K.C. (2007). Micro-organism inactivation during drying of small droplets or thin-layer slabs—A critical review of existing kinetics models and an appraisal of the drying rate dependent model. J. Food Eng..

[142] Friesen T., Hill G., Pugsley T., Holloway G., Zimmerman D. (2005). Experimental determination of viability loss of Penicillium bilaiae conidia during convective air-drying. Appl. Microbiol. Biotechnol..

[143] Spreutels L., Debaste F., Legros R., Haut B. (2013). Experimental characterization and modeling of Baker’s yeast pellet drying. Food Res. Int..

[144] Poirier I., Maréchal P.A., Gervais P. (1997). Effects of the kinetics of water potential variation on bacteria viability. J. Appl. Microbiol..

[145] Marechal P.A., de Marnañón I.M., Poirier I., Gervais P. (1999). The importance of the kinetics of application of physical stresses on the viability of microorganisms: Significance for minimal food processing. Trends Food Sci. Technol..

[146] Chandralekha A., Tavanandi A.H., Amrutha N., Hebbar H.U., Raghavarao K.S.M.S., Gadre R. (2016). Encapsulation of yeast (Saccharomyces cereviciae) by spray drying for extension of shelf life. Dry. Technol..

[147] Broeckx G., Vandenheuvel D., Claes I.J.J., Lebeer S., Kiekens F. (2016). Drying techniques of probiotic bacteria as an important step towards the development of novel pharmabiotics. Int. J. Pharm..

[148] Kumar A., Cincotti A., Aparicio S. (2020). Insights into the interaction between lipid bilayers and trehalose aqueous solutions. J. Mol. Liq..

[149] Ananta E., Volkert M., Knorr D. (2005). Cellular injuries and storage stability of spray-dried *Lactobacillus rhamnosus* GG. Int. Dairy J..

[150] Shu G., Li B., Dai C., Chen L., Yang X., Lei Z., Zhang M., Guo Y. (2022). Preparation of Saccharomyces boulardii powder by spray drying: Thermoprotectants optimization and stability evaluation. Prep. Biochem. Biotechnol..

[151] Sun H., Zhang M., Liu Y., Wang Y., Chen Y., Guan W., Li X., Wang Y. (2021). Improved viability of Lactobacillus plantarum embedded in whey protein concentrate/pullulan/trehalose hydrogel during freeze drying. Carbohydr. Polym..

[152] How Y.H., Teo M.Y.M., In L.L.A., Yeo S.K., Bhandari B., Pui L.P. (2023). Freeze drying of food-grade recombinant Lactococcus lactis NZ3900-fermented milk with different protecting agents. Int. Dairy J..

[153] Oluwatosin S.O., Tai S.L., Fagan-Endres M.A. (2022). Sucrose, maltodextrin and inulin efficacy as cryoprotectant, preservative and prebiotic—Towards a freeze dried *Lactobacillus plantarum* topical probiotic. Biotechnol. Rep..

[154] da Silva Guedes J., Pimentel T.C., Diniz-Silva H.T., Tayse da Cruz Almeida E., Tavares J.F., Leite de Souza E., Garcia E.F., Magnani M. (2019). Protective effects of *β*-glucan extracted from spent brewer yeast during freeze-drying, storage and exposure to simulated gastrointestinal conditions of probiotic lactobacilli. LWT.

[155] Stefanello R.F., Nabeshima E.H., Iamanaka B.T., Ludwig A., Fries L.L.M., Bernardi A.O., Copetti M.V. (2019). Survival and stability of Lactobacillus fermentum and Wickerhamomyces anomalus strains upon lyophilisation with different cryoprotectant agents. Food Res. Int..

[156] Zemančíková J., Kodedová M., Papoušková K., Sychrová H. (2018). Four Saccharomyces species differ in their tolerance to various stresses though they have similar basic physiological parameters. Folia Microbiol..

[157] Potts M. (1994). Desiccation tolerance of prokaryotes. Microbiol. Rev..

